# Hard X-ray phase-contrast-enhanced micro-CT for quantifying interfaces within brittle dense root-filling-restored human teeth

**DOI:** 10.1107/S1600577520005603

**Published:** 2020-05-20

**Authors:** Ana Prates Soares, Uwe Blunck, Kerstin Bitter, Sebastian Paris, Alexander Rack, Paul Zaslansky

**Affiliations:** a Department of Operative and Preventive Dentistry, Charité Universitätsmedizin Berlin, Aßmannshauser Straße 4-6, 14197 Berlin, Germany; b ESRF – The European Synchrotron, CS 40220, Grenoble Cedex 9, Grenoble 38043, France

**Keywords:** phase contrast enhanced micro-CT, interfacial gaps, restoration flaws, dental composites, resin bonding

## Abstract

Bonding of resin composite fillings, for example following root-canal treatment, is a challenge because remaining gaps grow and lead to failure. Here, phase-contrast-enhanced micro-computed tomography (PCE-CT) is used to explore methods of non-destructive quantification of the problem, so that countermeasures can be devised. Five human central incisors with damaged crowns were root-filled followed by restoration with a dental post. Thereafter, the crowns were rebuilt with a resin composite that was bonded conventionally to the tooth with a dental adhesive system (Futurabond U). Each sample was imaged by PCE-CT in a synchrotron facility (ID19, European Synchrotron Radiation Facility) with a pixel size of 650 nm. The reconstructed datasets from each sample were segmented and analysed in a semi-automated manner using *ImageJ*. PCE-CT at sub-micrometre resolution provided images with an impressive increased contrast and detail when compared with laboratory micro-computed tomography. The interface between the dental adhesive and the tooth was often strongly disrupted by the presence of large debonded gaps (on average 34% ± 15% on all surfaces). The thickness of the gaps spanned 2 µm to 16 µm. There was a large variability in the distribution of gaps within the bonding area in each sample, with some regions around the canal exhibiting up to 100% discontinuity. Although only several micrometres thick, the extensive wide gaps may serve as gateways to biofilm leakage, leading to failure of the restorations. They can also act as stress-raising ‘cracks’ that are likely to expand over time in response to cyclic mechanical loading as a consequence of mastication. The observations here show how PCE-CT can be used as a non-destructive quantitative tool for understanding and improving the performance of clinically used bonded dental restorations.

## Abstract 2

Phase-contrast enhanced micro-computed tomography reveals huge discontinuities at the interfaces between dental fillings and the tooth substrate. Despite the complex micromorphology, gaps in bonding could be visualized and quantified in 3D.

## 1. Introduction   

Extensive caries (tooth decay) causes large destruction of both crowns and roots in teeth. To fix this, the dentist restores the tooth shape and function using a combination of bonded composite materials that need to tightly adhere to the substrate. Biomaterials are typically chosen for mechanical durability and for aesthetic considerations, while matching the mechanical behaviour to the remaining tooth structure. Such dental biomaterials are used to return the tooth into function for patient satisfaction, and specifically to make sure the reconstruction withstands the repeating loads of mastication for many years. In fact, a main objective of contemporary dental treatment is to establish strong and continuous bonds between the filling and the tooth substrate. Current treatment protocols advocate bonded sealing for a variety of reasons. These include increased mechanical stability of the restoration due to an improved distribution and resistance to stresses, as well as prevention of bacterial percolation at the interface (Hayashi *et al.*, 2017 ▸).

Indeed nowadays clinicians have at their disposal a myriad of bonding systems produced by different manufacturers (Sofan *et al.*, 2017 ▸). All are able to bond composites to remaining tooth tissues. However, the resulting interactions between biomaterial and tooth structure are still not ideal (Van Meerbeek *et al.*, 2003 ▸) and often failure occurs due to discontinuities at the interface between the adhesive and tooth tissues. Such gaps are undesirable because fluids and bacteria may penetrate, impairing the longevity of the restorations (Van Meerbeek *et al.*, 2003 ▸). Discontinuities at the interfaces also act as stress-raising ‘cracks’ that are highly likely to expand over time in response to cyclic mechanical loading (as a consequence of mastication, see, for example, Zaslansky *et al.*, 2016 ▸). Propagation of such cracks along paths of minimal resistance, typically at the bond interface, will eventually result in dislodgement and failure of the restoration (Tay & Pashley, 2007 ▸).

The problem of tooth bonding in dentistry is not new. Much has changed since the early works of Buonocore (1955 ▸) who introduced pre-treatment of the dental substrate by use of phospho­ric acid etching. Such conditioning improves the chemical and mechanical attachment (Buonocore, 1955 ▸). This approach is as valid today as it was decades ago. Consequently, there are many routinely used products known as ‘etch-and-rinse’ dental bonding systems, for which phospho­ric acid etching and water rinsing is required prior to application of the adhesive. Alternative products have since emerged known as ‘self-etching’ systems that contain acidic monomers in their composition. Such ‘self-etching’ products can condition the tooth while forming a chemical bond (Van Meerbeek *et al.*, 2003 ▸). More recently, so-called ‘universal adhesives’ have appeared on the dental-materials market, and they can be applied in both ‘etch-and-rinse’ and ‘self-etching’ modes (Sofan *et al.*, 2017 ▸). Regardless of the bonding system used, the resulting interfaces with the tooth substrate are variable in quality and remain an active field of research, often due to uncontrollable voids and gaps within the bonded region.

**Figure 1:**
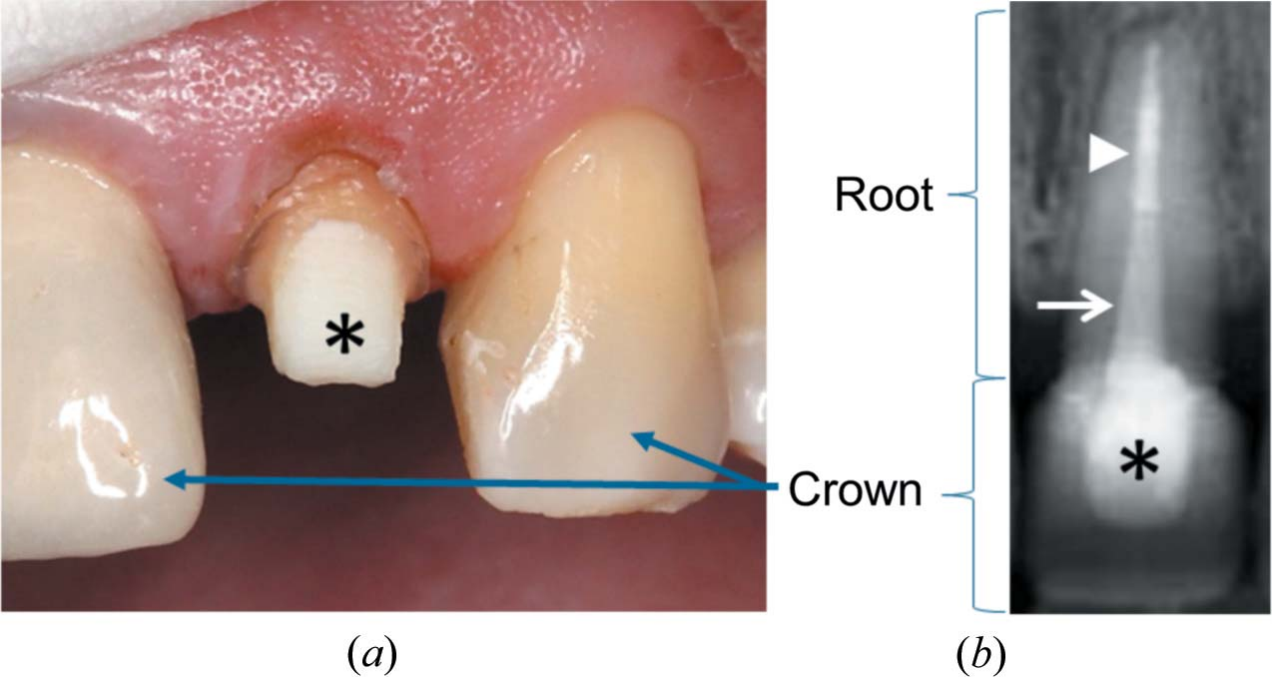
(*a*) Intra-oral photograph showing a tooth with a core (*), which is the only visible part of the post-and-core restoration during treatment, prior to coverage with a crown. (*b*) Radiograph of a similar tooth in which the core (*) is seen beneath an artificial crown, supported by a fibreglass post (white arrow) that is cemented into the disinfected and sealed (white arrowhead) root canal. (Clinical photographs courtesy of Drs Nestor Tzimpoulas and Wesley Thé, The Netherlands.)

Of the various types of dental treatments requiring bonding, the restoration of teeth and roots largely infected and destroyed by decay is the most complex. During treatment, the root canal system needs to be disinfected prior to tooth reconstruction. Disinfection followed by sealing of the tooth root are designed to stop and prevent re-infection, and the treatment is known as ‘root canal treatment’. Once the filling is completed, reconstruction of lost tooth structure must be planned. This usually requires a post-and-core restoration followed by crown construction. The post is used as a central pillar, and it is cemented into the root canal to support a composite material that replaces the bulk (core) of the crown (see example in Fig. 1 ▸). This is needed so that the post-and-core best retain a new artificial crown that is to be cemented permanently. In turn, the crown restoration provides the aesthetics and mechanical structure needed for efficient mastication. Finding materials that efficiently bond to the tooth may thus be considered essential for the long-term outcome of such restorations (Rasimick *et al.*, 2010 ▸).

For all the currently available materials on the market, establishing a continuous attachment between adhesive and tooth substrate is an ongoing challenge. It is thus important to be able to quantify the morphology and continuity of the bonded interfaces in the restored tooth, non-destructively. Indeed the quality of the bond produced depends on many factors ranging from the characteristics of the tooth surface (degree of mineralization, porosity, organic composition, *etc*.) to attributes of the bonding system (viscosity, wettability, acidity, *etc*.) (Perdigao, 2010 ▸; Cardoso *et al.*, 2011 ▸). Specifically, discontinuities at the interface known as ‘interfacial gaps’ are a major concern. Such gaps are usually studied by serial sectioning of the samples and microscopy imaging (Heintze, 2013 ▸), either with or without dye infiltration; dye is used to enhance the contrast of the gaps (*e.g.* organic colourants or silver nitrate) (Neves *et al.*, 2014 ▸). Overall, destructive methods such as slicing produce unwanted artefacts (Zaslansky *et al.*, 2011 ▸) whereas deep tracer infiltration may lead to ‘false-positive’ findings (Kriznar *et al.*, 2019 ▸; Shemesh *et al.*, 2008 ▸). Although 3D imaging techniques based on tomography are frequently used (*e.g.* Carrera *et al.*, 2015 ▸; Bakhsh *et al.*, 2011 ▸; Kwon & Park, 2012 ▸), the low density of polymer-based adhesive systems restricts the visibility of interfacial gaps using conventional X-ray imaging methods and hence it has been almost impossible to study the integrity of composite bonding (Bakhsh *et al.*, 2011 ▸).

Phase-contrast-enhanced micro-computed tomography (PCE-CT) accentuates interfaces due to the combined effects of high flux ‘partial-coherence’ X-rays. This facilitates the differentiation between materials with similar density (Cloetens *et al.*, 1996 ▸), in 3D, requiring no additives (*e.g.* dye) to increase contrast. 3D measurements of whole, root-treated teeth have previously been demonstrated using PCE-CT, revealing different density dental materials at micrometre resolution (Zaslansky *et al.*, 2011 ▸; Moinzadeh *et al.*, 2016 ▸; Soares *et al.*, 2019 ▸); however, application of these methods to help tackle bonded restoration is still missing. The aim of the present work is to outline a 3D quantitative approach to measure low-density inclusions at interfaces between tooth tissues and polymer-based dental adhesives, using PCE-CT in wet restored teeth.

## 2. Materials and methods   

### 2.1. Sample preparation   

For imaging the interfaces in restored teeth, samples were prepared using standardized clinically relevant protocols. These mimic the same working conditions followed in routine dental office procedures, as described below. For simplicity, all materials and chemicals used are listed in Table 1 ▸.

### 2.2. Root canal treatment   

Five human upper central incisors were obtained with written informed consent under an ethics-approved protocol (EA4/102/14) by the Ethical Review Committee of the Charité Universitätsmedizin Berlin, Germany, and were stored in an antiseptic solution prior to the experiment. Each tooth had its root canal treated following a standardized protocol (Soares *et al.*, 2019 ▸) by canal instrumentation and use of disinfecting irrigation followed by canal rinsing and drying. Each root canal was sealed with a standard root filling material. The filling comprised a gutta percha cone coated with a sealing cement which was vertically compacted into the canal.

### 2.3. Post cementation   

Following 24 h of sample storage (to allow the materials to set in a moist environment and at room temperature) each tooth was restored using a fibreglass dental post. This was cemented into the root canal with a self-adhesive resin cement, following manufacturers recommendations [for details, see Table 1 ▸ as well as Soares *et al.* (2019 ▸)]. Such dental posts are routinely used as pillars for the reconstruction of the tooth crown.

### 2.4. Crown restoration   

**Figure 2:**
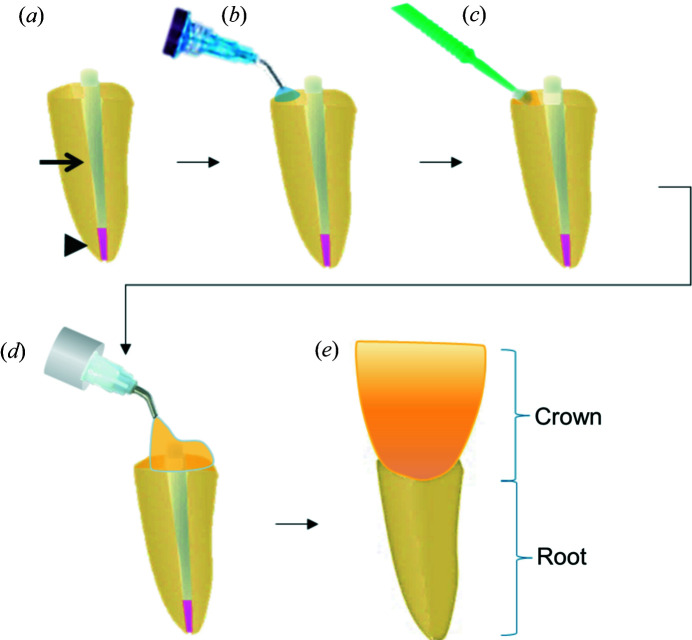
Typical workflow for root canal and crown restoration: (*a*) specimens after root canal treatment (seal – black arrowhead) and post (black arrow) cementation (*b*) receive acid-etching treatment of the restoration margins. The surface is then washed and briefly dried. (*c*) A universal dental adhesive (bonding system) is applied using dental brushes and light cured (photo-polymerized). (*d*) A dental resin composite is then placed on the post and on the exposed areas of the tooth to rebuild (*e*) the original crown shape.

Each tooth crown was restored immediately after post cementation. For that, the exposed tooth cervical area was acid etched for 10 s, followed by rinsing and air drying. A dental adhesive system was applied following manufacturers recommendations, including light curing for 10 s (Table 1 ▸, Fig. 2 ▸). A standard resin composite was placed over the adhesive layer and onto the dental post to rebuild the crown form. Following chemical polymerization (curing), the crown composite was light cured for 40 s to ensure full chemical activation and cross-linking. After 5 min, the resin was sufficiently hard and the material was trimmed and polished to reach the final shape of the crown.

### 2.5. Imaging and reconstruction   

After tooth restoration, each sample was mounted in a transparent vial (Micro tube 2 ml, Sarstedt, Nümbrecht, Germany), padded with wet foam to maintain humidity and to avoid dehydration during imaging.

**Figure 3:**
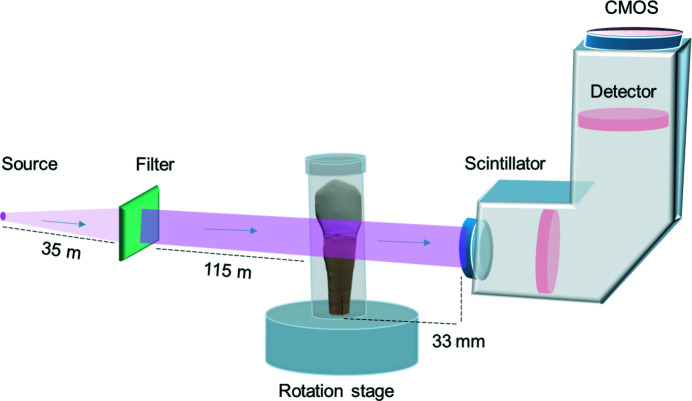
Schematic illustration of the experiment showing the incoming X-ray beam (source is far to the left, not drawn to scale) passing through relevant filters to reach the sample mounted on the rotation stage. The samples were kept humid in a sealed plastic vial throughout the experiment. Fresnel propagation was induced by increasing the distance between the sample and detector (33 mm) leading to edge enhancement with sufficient contrast to view the adhesive layer (Cloetens *et al.*, 1996 ▸).

Laboratory micro-computed tomography (µCT) (Skyscan 1172; Bruker micro CT, Kontich, Belgium) was used to first image each specimen (16 µm pixel size, 700 ms exposure time). Following reconstruction (NRecon 1.7.1.0; Bruker micro CT, Kontich, Belgium) the architectures of the restorations were examined in both 2D and 3D (*ImageJ* 1.52d, National Institute of Health, USA; Amira ZIB-Edition, Konrad-Zuse-Zentrum für Informationstechnik Berlin, Germany). The cervical areas, including the rim between root and the crown restoration were selected for imaging by PCE-CT in a synchrotron. Each sample was scanned on beamline ID19 of the European Synchrotron Radiation Facility (ESRF, Grenoble, France) using inline propagation-based contrast microtomography (Fig. 3 ▸). An X-ray photon energy of 34 keV was used with a pco.edge camera (PCO AG, Kelheim, Germany), and an LSO:Tb scintillator in a custom-made imaging system (OptiquePeter, Lentilly, France) with an effective pixel size of 650 nm. To enhance the visibility of gaps, PCE-CT scans were obtained using a sample-to-detector distance of 33 mm. Each sample was mounted on the high-resolution rotation stage and a total of 4900 radiographic projections were recorded (200 ms exposure times) while continuously rotating the samples by 360°. To accommodate the larger field of view, we used a horizontal stitching mode to fully image samples that were wider than a single frame of the camera. ESRF in-house code was used to reconstruct the data, enhancing contrast in the radiographs by means of a modified Paganin-based filtering (δ/β ratio of 200; Mirone *et al.*, 2014 ▸).

### 2.6. Image analysis   

**Figure 4:**
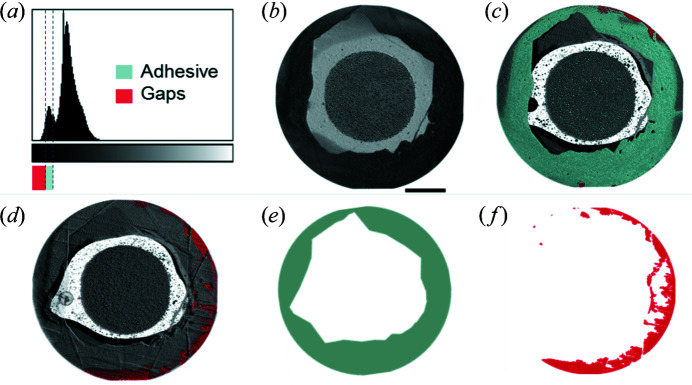
Proposed data processing pipeline for bonded restoration analysis of typical 3D reconstructed PCE-CT datasets: (*a*) histograms of the full volumes (*b*) were used to binarize and segment the interfacial gaps (grey value range matching the interfacial gap region is marked light blue beneath the graph abscissa). (*c*) The adhesive layer, marked light blue – compare panel (*e*) – is well defined. (*d*) Gaps at the interface (red points on the 2D slice) are also identified based on the histogram [red marked region corresponding to gaps in (*a*)]. The area of bonding between tooth and adhesive (*e*) was determined (see additional details in the text), from which the percentage of interfacial gaps (*f*) was calculated as the ratio between (*f*) and (*e*). Scale bar: 500 µm.

**Figure 5:**
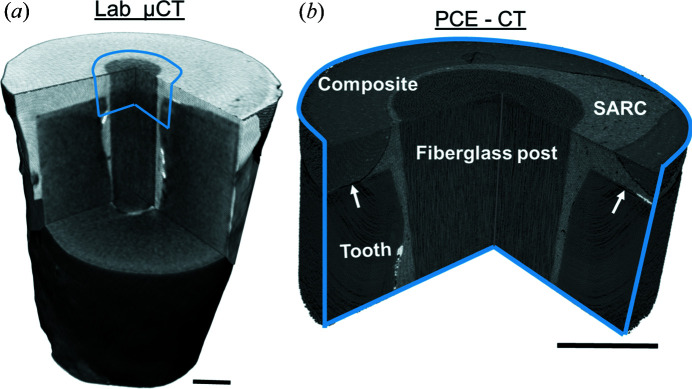
(*a*) Laboratory µCT images of the entire root length and a part of the crown lacking sufficient contrast to show the difference between some of the materials or to depict gaps at their interfaces. (*b*) PCE-CT revealed extensive details in the structure of the restoration, showing the morphology of the tooth and the presence of the adhesive layer (white arrows), as well as details within and the difference between the fibreglass post, self-adhesive resin cement (SARC) and the resin composite of the crown. Scale bars: 500 µm.

**Figure 6:**
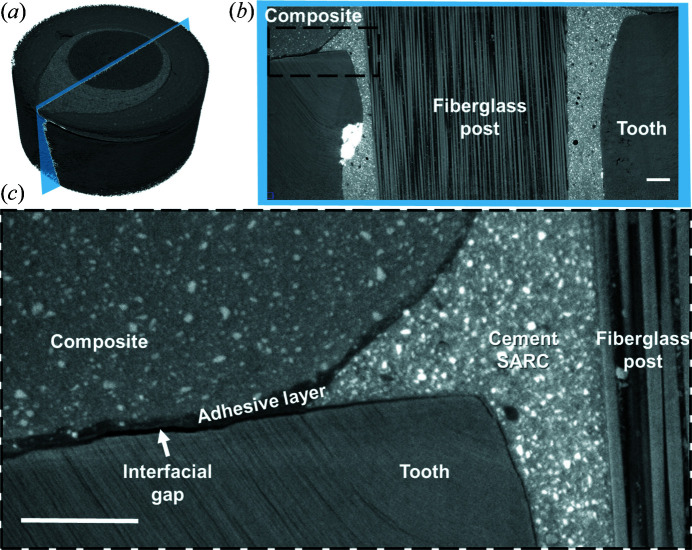
Full volumes (*a*) were rendered in 3D where longitudinal slices were used to verify the difference in contrast between composite, fibreglass post, SARC, tooth and adhesive layer (*b*) and (*c*). Note the presence of an interfacial gap between tooth and adhesive layer [(*c*) – white arrow] and the details appearing in the magnified inset. Scale bars: 100 µm.

**Figure 7:**
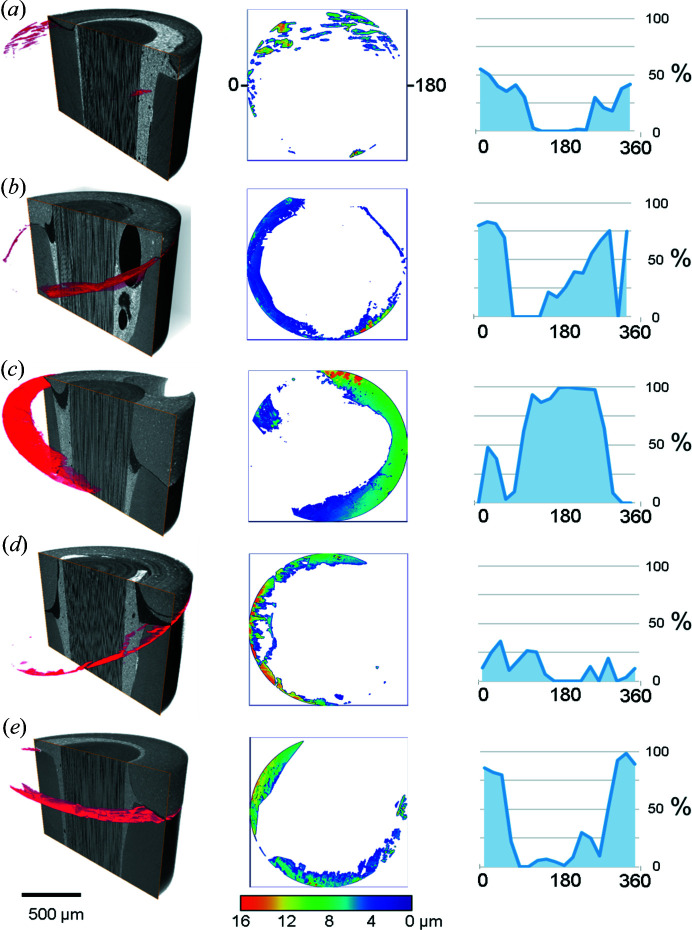
3D renderings of five samples (on the left), showing longitudinally sliced volumes portraying tooth and restoration materials virtually cropped (grey), on which the interfacial gaps between dentin and adhesive layer are depicted in 3D (red). Thickness maps (middle) of the interfacial gaps show a spatially varying distribution around the imaged region. The calibration bar represents the thickness of the gaps. Quantitative evaluation of the percentage of gaps measured in 18 segments around the axis of the post reveals strong orientation variation with certain regions of debonding exceeding 80% of the surface area.

The high-resolution reconstructed datasets characteristically contained about 2100 slices orthogonal to the long axis of the tooth. Each dataset occupies 80 Gigabytes or more of disk space. Data were visualized using *ImageJ* and *Amira*. Typical 2D slices are shown in Fig. 4 ▸ and 3D renderings of the reconstructions are shown in Figs. 5–7. Due to phase contrast enhancement at discontinuities and between different density components, details of the post, cement, voids and tooth tissues are clearly visible.

To isolate debonding and interfacial gaps, the datasets were processed using the free extension package *MorpholibJ* for *ImageJ* (Legland *et al.*, 2016 ▸). As a first step, a threshold was defined using the grey value range corresponding to gaps in the restoration [Fig. 4 ▸(*a*)]. The resulting binary images had their different sets of connected pixels and voxels individually numbered and labelled using the ‘Connected components Labelling’ algorithm. The different components localized to the adhesive layer were then visually selected, discarding the irrelevant (outside of the interface) labels by using the ‘Select Labels’ function of *MorpholibJ*. The final Boolean volume comprising only the interfacial gaps between adhesive and tooth was overlaid onto the original volume (Fig. 5 ▸) to verify successful segmentation (Fig. 6 ▸). The extracted volumes of voids were then further processed as follows: the thickness of the gaps was determined using the ‘Local thickness’ algorithm of *ImageJ* (Dougherty & Kunzelmann, 2007 ▸) (Fig. 7 ▸). The gap cross-sectional areas were determined by axially projecting the minimal intensity of the segmented volume along the tooth/post axis. Similarly, the tooth area conditioned for adhesive bonding was determined by projecting the maximum intensity of the full volume, thereafter thresholding it to separate the darker (conditioned tooth area) from the brighter (cement-covered tooth area) areas. The star-like appearance, as demonstrated in Fig. 4 ▸(*b*), is due to the cutting lines, used to remove excess soft cement during restoration construction: a sharp straight scalpel was used to clear overflow prior to crown construction. Thus PCE-CT even reveals laying steps during restoration fabrication. With 2D slices containing the conditioned tooth area and gap, the percentage of gaps at the interface was calculated. Radial, orientation-specific variations in the non-bonded regions were quantified to assess the distributions of gaps at the interface. With the centre of the restoration/post defined as a pivot, the tooth and gap areas were divided in 18 equal segments (sectors) spanning 360° around the tooth long axis, each extending 20° on the tooth surface. For each sector, the ratio of gaps to the area of bonding was quantified and plotted against the azimuthal angular axis.

## 3. Results   

Laboratory µCT scans generated datasets that reproduced the approximate geometry and the main components of the tooth restoration within the root and the reconstructed crown. In such data, however, there was no difference in contrast between the resin composites used for crown reconstruction and the resin cement (Fig. 5 ▸), nor was it possible to distinguish the adhesive polymer layer or any gaps therein. Furthermore, the interfaces between restorative materials and tooth substrate were blurred, making it impossible to evaluate the extent of bonding between the tooth tissues and the dental materials.

PCE-CT at sub-micrometre resolution provided 3D datasets with an impressive contrast and a remarkable amount of detail (Figs. 5–7). The micromorphology of the tooth and restoration were fully visible, revealing micrometre-diameter dentin tubules, highlighting the presence of fillers in the different resin composites and well reproducing the layout of single fibres within the fibreglass post. Example overviews and slices in the datasets are shown in a longitudinal slice in Fig. 6 ▸.

The enhanced contrast brought about with PCE-CT highlights gaps between the adhesive (Fig. 6 ▸), tooth substrate and resin composite. Although significant areas of the interface appeared to be well bonded and continuous, interfacial gaps were surprisingly extensive and well identifiable. The thickness of the gaps (*e.g.* Fig. 6 ▸, white arrow) varied from 2 µm to 16 µm and adversely affected an average of 34% (±15%) of the contact surfaces between dentin and adhesive, as demonstrated here in five different teeth.

The results from the analysis of 18 sections of each sample, shown in the graphs of Fig. 7 ▸, reveal the large variability in the percentage of gaps between different sides of the same restoration in different samples. There were specimens with extreme disparities, where some segments had no gaps at the tooth-restoration interface, while others exceeded 95% of gaps. Such regions have a poor bond between the restoration and tooth tissue. The graphs highlight the variability in the total percentage of gaps between samples. Note that the interface is not a plane but is a 3D surface such that proper quantification required 3D sub-micrometre high-contrast information that is obtained non-destructively. PCE-CT provided this information, which is very different and complementary to the information from physical slicing. The latter is particularly difficult to achieve in the brittle tooth-filling interface in different directions, which may then under-represent the full extent of the problem for any given filling.

## 4. Discussion   

The present work provides a proof-of-principle feasibility test resolving the challenges involved in imaging and quantifying bonding between teeth and restorations. This has great significance for developing and validating the achievement of reliable, gap-free bonded margins. With PCE-CT it is possible to quantify and compare thin gaps (<20 µm-thick) at the interfaces in adhesively restored human teeth. Of great concern is the observation that in each tooth at least 50% of the visible section of the outer imaged rim exhibited some form of gap, as seen in the central column of Fig. 7 ▸. Achieving a complete, continuous gap-free bond in real restored teeth thus remains a challenge. The samples treated here used a material belonging to the latest generation of adhesives (Futurabond U) where ∼35% of the bonded tooth surfaces exhibited discontinuous interfacial gaps. The distribution of gaps (both extent and orientation) was highly variable both within and between samples, which is typical for biomedical samples. This is an additional reason why non-destructive testing is needed for quantification and finding solutions to polymer bonding. Despite following strict protocols and treating the tested teeth under ideal, identical conditions *in vitro*, it is extremely difficult to produce a predictable sealed bonded interface. From a biological standpoint, establishing a continuous interface between tooth and adhesive is a major objective of treatment. It is a clinical objective aimed at preventing damages associated with water sorption, infiltration of bacteria and bacterial by-products, reportedly associated with secondary caries and/or re-infection of the root canal system (Van Meerbeek *et al.*, 2003 ▸). From a biomechanical standpoint, structural gaps act as stress-raisers with the result that any bending that pulls against the bonded interface results in high stress concentration. This leads to propagation of the debonded region (adhesive failure) or to propagation of cracks into the materials forming the bond (cohesive failure) (Tay & Pashley, 2007 ▸; Chen *et al.*, 2015 ▸). This is of particular concern for bonded surfaces following root canal treatment, since mechanical fatigue is very likely to occur due to structural defects beneath the restored crown used for mastication. Indeed cyclic loading is typical for teeth and restorations that must function for many years. In the long term, debonding and expansion of interfacial gaps may lead to complete detachment and failure of the tooth/restoration.

Due to the lack of contrast and resolution in the laboratory µCT images, it was not possible to observe the adhesive layer or the interfaces between materials. PCE-CT resolved this problem as it is extremely effective and may be the only possible means to non-destructively image interfacial gaps between the low-density polymer material and other structures within the tooth restorations (Soares *et al.*, 2019 ▸). Visualizing discontinuities at the interfaces of a low-absorption dental adhesive with µCT is challenging (Mollica *et al.*, 2004 ▸; De Santis *et al.*, 2005 ▸; Kriznar *et al.*, 2019 ▸; Neves *et al.*, 2014 ▸; Carrera *et al.*, 2015 ▸). The lack of contrast between materials, unavailability of sufficient resolution and the accumulation of artefacts inherent to synchrotron-based imaging complicates the process of quantifying interfacial gaps in 3D (Kriznar *et al.*, 2019 ▸; Neves *et al.*, 2014 ▸). Previous work attempted to enhance contrast by increasing the absorption of restorative materials, adding radiopaque fillers to their composition (Rominu *et al.*, 2014 ▸). Other authors limited the use of PCE-CT to image high-absorption adhesives (rich in radiopaque particles), while applying thick layers of adhesive to the samples (Kriznar *et al.*, 2019 ▸). The adhesive used in our study has no fillers and was applied in one single layer, as recommended by the manufacturer for clinical use. Moreover, as can be seen in Fig. 6 ▸, crisp images with high signal-to-noise ratios were acquired from intact, hydrated tooth samples that were root canal treated and restored. All these make the use of PCE-CT of great interest to communities interested in improving polymer-based bonding.

Discontinuities at the interface between restorative materials and tooth substrate have been reported using high-contrast dyes such as silver nitrate. This radiopaque liquid has been widely used in dental research due to its high contrast in radiographs (Mollica *et al.*, 2004 ▸; De Santis *et al.*, 2005 ▸; Kriznar *et al.*, 2019 ▸; Neves *et al.*, 2014 ▸; Carrera *et al.*, 2015 ▸). However, it is unclear which and what dimensions of gaps are sufficiently accessible to allow penetration and visualization of the dye. Indeed, infiltration of silver nitrate into gaps in tooth specimens was shown to be suboptimal, resulting in either the underestimation or overestimation of the sizes of interfacial gaps (addressed by Kriznar *et al.*, 2019 ▸). Overestimation may occur when silver nitrate accumulates in the naturally porous morphology of the tooth. The use of PCE-CT eliminates the need to enhance contrast using any dye with the result that the location, thickness, extension and even volume of interfacial gaps can be more accurately assessed.

The use of semi-automated image segmentation presented here will benefit from further improvements but already has many advantages. Since the data are obtained non-destructively, effects of artefacts and filtering can be tested and the 3D data can be quantified in a repeatable, quantitative operator-independent way (Carrera *et al.*, 2015 ▸). This provides information at a higher dimension that better represents the spatial arrangement of materials as compared with examining 2D physical sections. The methodology described here demonstrates the importance and feasibility of analysing intact, hydrated, as-prepared human-tooth samples in 3D, circumventing the need for cutting the tooth for direct visualization. Traditional approaches to investigate interfacial gaps require sample cross-sectioning and microscopic measurements. Although informative, such approaches are destructive, and may inadvertently cause the underestimation of gaps due to accumulation of tooth tissue (smear layer) brought about by sample polishing or overestimation of gaps as a consequence of sample dehydration (Zaslansky *et al.*, 2011 ▸). Cutting and dehydration of samples for microscopic assessment, especially with scanning electron microscopes, can also lead to cracks and overestimation of gaps near the interface, due to the brittleness of the samples (Soares *et al.*, 2019 ▸). Furthermore, as shown with the examination of 18 segments around each tooth, there can be great variability in the percentage of debonding within any single sample. This observation would translate into contrasting statistics of the data if the samples were simply sliced in different directions and examined in 2D. PCE-CT, especially at energies above 30 keV, resolves all this if sufficient access to beamlines is made available. This is augmented by the fact that the samples can be imaged under humid conditions – avoiding dehydration-induced cracking (Shemesh *et al.*, 2018 ▸), hence resembling oral conditions.

Synchrotron µCT imaging is an effective tool for examining different biological samples. The high photon flux density and almost parallel beams with a wide range of energies is suited for imaging both low- and high-density structures. Phase-contrast imaging (Cloetens *et al.*, 1996 ▸) is about three orders of magnitude more sensitive to density differences than attenuation-based methods (Lewis, 2004 ▸). Additionally, the acquisition time for tomographic imaging with similar resolution to other techniques is 10 to 100 times faster (Betz *et al.*, 2007 ▸). This enables researchers to swiftly image a large range of biomaterials inside the tooth structure (Mollica *et al.*, 2004 ▸; De Santis *et al.*, 2005 ▸; Zaslansky *et al.*, 2011 ▸; Rominu *et al.*, 2014 ▸; Hedayat *et al.*, 2016 ▸; Fatima *et al.*, 2016 ▸; Moinzadeh *et al.*, 2016 ▸; Kriznar *et al.*, 2019 ▸). Although in the experiments reported here the field of view was restricted to the centre of the specimen, additional overlapping imaging or future developments in next generation facilities will make it possible to image entire tooth crowns faster and at even higher resolutions. This will provide more information about different interactions between material and substrate. Faster acquisition rates amounting to terabytes of reconstructed data will require further developments of data storage, processing and analysis. The large number of virtual slices generated for each dataset from synchrotron µCT makes manual (slice-by-slice) segmentation impractical (Meneses *et al.*, 2011 ▸). The present work showcases one tested and tried approach to analyse full volumes containing interfacial gaps in restored human teeth. Although fairly simple, it is time consuming and requires extensive computation. It is also not applicable for the clinical setting. Future developments in the field of artificial intelligence and machine learning will hopefully improve image segmentation automation (Dimiduk *et al.*, 2018 ▸; Beliaev *et al.*, 2020 ▸).

The adhesive used for testing here was Futurabond U, a clinically used universal adhesive system. It can be deployed either with or without prior acid etching of the tooth substrate (Sofan *et al.*, 2017 ▸). In the current work, phospho­ric acid was used to etch the tooth substrate (dentin) before adhesive application, since the literature shows that it results in a higher bond strength between material and tooth (Cengiz & Ünal, 2019 ▸). Futurabond U has a clinically favourable *in vitro* performance record, comparable with other universal adhesive systems (Chen *et al.*, 2015 ▸) and is thus excellent for clinical use, like many other similar products. The investigation of gaps at the interface of different adhesives may add to the data needed to improve the performance and connection between materials and substrate. Furthermore, future developments may make it possible to correlate between observations of interfacial gaps and the important effects of bond strength (Brito-Junior *et al.*, 2015 ▸; Irie *et al.*, 2010 ▸). Greater availability of PCE-CT will pave the way to assess a large number of samples that can be mechanically loaded and imaged again, to observe debonding dynamics. Such studies can broaden understanding and enable testing of novel approaches to improve interactions between adhesives and the dental substrate.

## 5. Conclusions   

The present work provides details about steps needed to measure gaps and the areas that they affect, without the use of any tracer dye and while strictly adhering to clinically used materials and procedures allowing reproducibility and comparability. The observations here clearly establish the use of PCE-CT for advancing our understanding about the ability of clinically used dental adhesive systems to form continuous interfaces with tooth tissues when measured *in vitro*. It is also a valuable tool for imaging interfacial gaps between thin polymer adhesives and tooth substrate in restored hydrated, treated teeth in 3D. Flaws in the thin polymer bonding layer were quantified non-destructively. Although a possible quantitative data processing pipeline was proposed, much more can be done for improved image segmentation and analysis. The use of PCE-CT will assist efforts to systematically assess the integrity of contact between different clinically used dental materials.

## Figures and Tables

**Table 1:** Materials and descriptions of their use Materials and chemicals used for sample preparation are in the order of appearance in the text.

Description	Nomenclature	Manufacturer
Storage antiseptic solution	0.5% chloramine T	Merck KGaA, Darmstadt, Germany
Canal instrumentation files	Endodontic nickel titanium files	X1-X4 Pro Taper Next System, Dentsply Sirona, Ballaigues, Switzerland
Root canal disinfection solution	1% sodium hypochlorite solution	Hedinger GmbH, Stuttgart, Germany
Root canal rinsing solution	Saline solution	Braun, Melsungen, Germany
Root canal drying material	Sterilized paper filter cones	Pro Taper Next System X4 Paper Points Dentsply Sirona
Root canal sealing cone	Gutta percha	Pro Taper Next System X4 gutta percha cone, Dentsply Sirona
Root canal sealing cement	Dual paste ep­oxy based sealing cement	AH Plus, Dentsply Sirona
Vertical compaction of canal seal	Root canal obturation unit	Calamus Dual System, Dentsply Sirona
Dental post	Fibreglass post	Dentin Post size 090, Komet, Lemgo, Germany
Resin cement	Dual curing, self-adhesive resin cement	RelyX Unicem 2, Automix delivery system, 3M ESPE Dental Products, St Paul, USA
Acid etch	Phospho­ric acid at 37%	Orbi Flow, Orbis Dental, Münster, Germany
Dental adhesive system	Universal dual-curing adhesive system	Futurabond U Single Dose, Voco, Cuxhaven, Germany (LOT No. 1601051)
Light cure	LED light curing device	Valo Corded, Ultradent Products Inc, South Jordan, USA
Resin composite	Core build up dual curing resin composite	Rebilda DC, Voco

## References

[bb2] Bakhsh, T. A., Sadr, A., Shimada, Y., Tagami, J. & Sumi, Y. (2011). *Dent. Mater.* **27**, 915–925. 10.1016/j.dental.2011.05.003 21665263

[bb3] Beliaev, M., Zöllner, D., Pacureanu, A., Zaslansky, P., Bertinetti, L. & Zlotnikov, I. (2020). *J. Struct. Biol.* **209**, 107432. 10.1016/j.jsb.2019.107432 31816415

[bb4] Betz, O., Wegst, U., Weide, D., Heethoff, M., Helfen, L., Lee, W. & Cloetens, P. (2007). *J. Microsc.* **227**, 51–71. 10.1111/j.1365-2818.2007.01785.x 17635659

[bb5] Brito-Junior, M., Leoni, G. B., Pereira, R. D., Faria-e-Silva, A. L., Gomes, E. A., Silva-Sousa, Y. T. & Sousa-Neto, M. D. (2015). *J. Endod.* **41**, 2058–2063. 10.1016/j.joen.2015.09.009 26602449

[bb51] Buonocore, M. G. (1955). *J. Dent. Res.* **34**, 849–853. 10.1177/00220345550340060801 13271655

[bb6] Cardoso, M. V., de Almeida Neves, A., Mine, A., Coutinho, E., Van Landuyt, K., De Munck, J. & Van Meerbeek, B. (2011). *Aust. Dent. J.* **56**, 31–44. 10.1111/j.1834-7819.2011.01294.x 21564114

[bb7] Carrera, C. A., Lan, C., Escobar-Sanabria, D., Li, Y., Rudney, J., Aparicio, C. & Fok, A. (2015). *Dent. Mater.* **31**, 382–390. 10.1016/j.dental.2015.01.002 PMC4374050 25649496

[bb8] Cengiz, T. & Ünal, M. (2019). *Microsc. Res. Tech.* **82**, 1032–1040. 10.1002/jemt.23250 30866096

[bb50] Chen, C., Niu, L. N., Xie, H., Zhang, Z. Y., Zhou, L. Q., Jiao, K., Chen, J. H., Pashley, D. H. & Tay, F. R. (2015). *J. Dent.* **43**, 525–536. 10.1016/j.jdent.2015.03.004 25797702

[bb1] Cloetens, P., Barrett, R., Baruchel, J., Guigay, J.-P. & Schlenker, M. (1996). *J. Phys. D Appl. Phys.* **29**, 133–146.

[bb11] De Santis, R., Mollica, F., Prisco, D., Rengo, S., Ambrosio, L. & Nicolais, L. (2005). *Biomaterials*, **26**, 257–270. 10.1016/j.biomaterials.2004.02.024 15262468

[bb13] Dimiduk, D. M., Holm, E. A. & Niezgoda, S. R. (2018). *Integr. Mater. Manuf. Innov.* **7**, 157–172.

[bb14] Dougherty, R. P. & Kunzelmann, K. H. (2007). *Microsc. Microanal.* **13**, 1678–1679.

[bb15] Fatima, A., Kulkarni, V. K., Banda, N. R., Agrawal, A. K., Singh, B., Sarkar, P. S., Tripathi, S., Shripathi, T., Kashyap, Y. & Sinha, A. (2016). *J. X-ray Sci. Technol.* **24**, 119–132. 10.3233/XST-160530 26890899

[bb16] Hayashi, J., Shimada, Y., Tagami, J., Sumi, Y. & Sadr, A. (2017). *J. Dent. Res.* **96**, 992–998. 10.1177/0022034517709005 28521113

[bb17] Hedayat, A., Nagy, N., Packota, G., Monteith, J., Allen, D., Wysokinski, T. & Zhu, N. (2016). *J. Synchrotron Rad.* **23**, 777–782. 10.1107/S1600577516002198 27140158

[bb18] Heintze, S. D. (2013). *Dent. Mater.* **29**, 59–84. 10.1016/j.dental.2012.07.158 22920539

[bb19] Irie, M., Maruo, Y., Nishigawa, G., Suzuki, K. & Watts, D. C. (2010). *Dent. Mater.* **26**, 608–615. 10.1016/j.dental.2010.02.012 20334906

[bb20] Kriznar, I., Zanini, F. & Fidler, A. (2019). *Clin. Oral Investig.* **23**, 2371–2381. 10.1007/s00784-018-2680-y 30302609

[bb21] Kwon, O.-H. & Park, S.-H. (2012). *Restor. Dent. Endod.* **37**,41–49.

[bb22] Legland, D., Arganda-Carreras, I. & Andrey, P. (2016). *Bioinformatics*, **32**, 3532–3534. 10.1093/bioinformatics/btw413 27412086

[bb23] Lewis, R. A. (2004). *Phys. Med. Biol.* **49**, 3573. 10.1088/0031-9155/49/16/005 15446788

[bb24] Meneses, A. A. M., Giusti, A., Almeida, A. P., Nogueira, L. P., Braz, D., Barroso, R. C. & de Almeida, C. E. (2011). *Nucl. Instrum. Methods Phys. Res. A*, **660**, 121–129.

[bb52] Mirone, A., Brun, E., Gouillart, E., Tafforeau, P. & Kieffer, J. (2014). *Nucl. Instrum. Methods Phys. Res. B*, **324**, 41–48.

[bb25] Moinzadeh, A. T., Farack, L., Wilde, F., Shemesh, H. & Zaslansky, P. (2016). *J. Endod.* **42**, 776–781. 10.1016/j.joen.2016.01.023 26994599

[bb26] Mollica, F., De Santis, R., Ambrosio, L., Nicolais, L., Prisco, D. & Rengo, S. (2004). *J. Mater. Sci. Mater. Med.* **15**, 485–492. 10.1023/b:jmsm.0000021125.40282.ba 15332622

[bb27] Neves, A. A., Jaecques, S., Van Ende, A., Cardoso, M. V., Coutinho, E., Lührs AK Zicari, F. & Van Meerbeek, B. (2014). *Dent. Mater.* **30**, 799–807. 10.1016/j.dental.2014.05.003 24908617

[bb28] Perdigao, J. (2010). *Dent. Mater.* **26**, e24–e37. 10.1016/j.dental.2009.11.149 20005565

[bb29] Rasimick, B. J., Wan, J., Musikant, B. L. & Deutsch, A. S. (2010). *J. Prosthodont.* **19**, 639–646. 10.1111/j.1532-849X.2010.00647.x 21040098

[bb30] Rominu, M., Manescu, A., Sinescu, C., Negrutiu, M. L., Topala, F., Rominu, R. O., Bradu, A., Jackson, D. A., Giuliani, A. & Podoleanu, A. G. (2014). *Dent. Mater.* **30**, 417–423. 10.1016/j.dental.2014.01.004 24530139

[bb31] Shemesh, H., Lindtner, T., Portoles, C. A. & Zaslansky, P. (2018). *J. Endod.* **44**, 120–125. 10.1016/j.joen.2017.07.025 29079053

[bb32] Shemesh, H., Souza, E. M., Wu, M. K. & Wesselink, P. R. (2008). *Int. Endod. J.* **41**, 869–872. 10.1111/j.1365-2591.2008.01440.x 18699787

[bb33] Soares, A. P., Bitter, K., Lagrange, A., Rack, A., Shemesh, H. & Zaslansky, P. (2019). *Int. Endod. J.* **53**, 392–402. 10.1111/iej.13232 31587321

[bb34] Sofan, E., Sofan, A., Palaia, G., Tenore, G., Romeo, U. & Migliau, G. (2017). *Ann. Stomatol. (Roma)*, **8**, 1–17. 10.11138/ads/2017.8.1.001 PMC5507161 28736601

[bb35] Tay, F. R. & Pashley, D. H. (2007). *J. Endod.* **33**, 391–398. 10.1016/j.joen.2006.10.009 PMC2223075 17368325

[bb12] Van Meerbeek, B., De Munck, J., Yoshida, Y., Inoue, S., Vargas, M., Vijay, P., Van Landuyt, K., Lambrechts, P. & Vanherle, G. (2003). *Oper. Dent.* **28**, 1–20. 12760693

[bb36] Zaslansky, P., Currey, J. D. & Fleck, C. (2016). *Bioinspir. Biomim.* **11**, 051003. 10.1088/1748-3190/11/5/051003 27615450

[bb37] Zaslansky, P., Fratzl, P., Rack, A., Wu, M. K. & Wesselink, P. R. (2011). *Int. Endod. J.* **44**, 395–401. 10.1111/j.1365-2591.2010.01830.x 21219359

